# The Effectiveness of Behavioral Interventions in Adults with Post-Traumatic Stress Disorder during Clinical Rehabilitation: A Rapid Review

**DOI:** 10.3390/ijerph19127514

**Published:** 2022-06-19

**Authors:** Francesca Gimigliano, Vanessa M. Young, Chiara Arienti, Silvia Bargeri, Greta Castellini, Silvia Gianola, Stefano G. Lazzarini, Antimo Moretti, Allen W. Heinemann, Stefano Negrini

**Affiliations:** 1 Department of Mental and Physical Health and Preventive Medicine, University of Campania Luigi Vanvitelli, 80138 Naples, Italy; francescagimigliano@gmail.com; 2 School of Social and Behavioral Sciences, Arizona State University, Phoenix, AZ 85051, USA; 3 Istituto di Ricovero e Cura a Carattere Scientifico (IRCCS), Fondazione Don Carlo Gnocchi, 20148 Milan, Italy; carienti@dongnocchi.it (C.A.); slazzarini@dongnocchi.it (S.G.L.); 4 Unit of Clinical Epidemiology, Istituto di Ricovero e Cura a Carattere Scientifico (IRCCS), Istituto Ortopedico Galeazzi, 20161 Milan, Italy; silvia.bargeri@grupposandonato.it (S.B.); greta.castellini@grupposandonato.it (G.C.); silvia.gianola@grupposandonato.it (S.G.); 5 Department of Medical and Surgical Specialties and Dentistry, University of Campania Luigi Vanvitelli, 80138 Naples, Italy; antimomor83@hotmail.it; 6 Shirley Ryan AbilityLab, Department of Physical Medicine and Rehabilitation, Feinberg School of Medicine, Northwestern University, Chicago, IL 60611, USA; aheinemann@sralab.org; 7 Department of Biomedical, Surgical and Dental Sciences, University La Statale, 20122 Milan, Italy; stefano.negrini@unimi.it; 8 Laboratory of Evidence-Based Rehabilitation, IRCCS Istituto Ortopedico Galeazzi, 20161 Milan, Italy

**Keywords:** PTSD, medical trauma, behavioral interventions, injury, rehabilitation

## Abstract

Background: This review examined the effectiveness of behavioral interventions for adults with post-traumatic stress disorder (PTSD) triggered by physical injury or medical trauma. It discusses implications in support of rehabilitation management for COVID-19 survivors diagnosed with PTSD. Methods: This study adhered to the Preferred Reporting Items for Systematic Reviews and Meta-Analyses guidelines and the Interim Guidance from the Cochrane Rapid Reviews Methods Group. The authors searched for randomized control trials in PubMed, Embase, and CENTRAL databases up to 31 March 2021. Results: Five studies (*n* = 459) met the inclusion criteria. Each study measured a different comparison of interventions. The certainty of the evidence was judged to be very low for all outcomes. Post-traumatic stress disorder symptom reduction was found to be in favor of trauma-focused cognitive-behavioral therapy, cognitive therapy, and cognitive-behavioral therapy. Cognitive function improvements were observed in favor of the cognitive processing therapy control intervention. Conclusions: Overall, there is uncertainty about whether behavioral interventions are effective in reducing PTSD symptoms and improving functioning and quality of life when the disorder is triggered by a physical or medical trauma rather than a psychological trauma. Further research should investigate their efficacy in the context of rehabilitation management and gather evidence on this population.

## 1. Introduction

The coronavirus disease 2019 (COVID-19) pandemic has challenged medical practices worldwide. Considering the unmet need for updated evidence for COVID-19 rehabilitation management, the World Health Organization Rehabilitation Program has prompted Cochrane Rehabilitation to conduct reviews of available evidence relevant to COVID-19–related illnesses to improve rehabilitation services in light of the pandemic [1]. Life-threatening diseases such as COVID-19 can trigger many mental-health-related problems such as depression, anxiety, and post-traumatic disorder (PTSD). When untreated, these disorders can negatively impact rehabilitation outcomes [2]. The complex response to medical trauma can be further explored through a biopsychological perspective and the application of such evidence is critical to multidisciplinary rehabilitation to support survivors of life-threatening diseases such as COVID-19.

The list of relevant health conditions requiring attention includes PTSD. PTSD is a mental health condition triggered by a harrowing experience, and it can affect individuals of any age. PTSD has increasingly been examined in the context of physical trauma, such as sudden injury or illness, including cardiovascular disease, motor vehicle injury, brain or spinal cord injury, traumatic childbirth, physical and sexual assault, intensive care unit survival [3,4,5], and—most recently—COVID-19 [6,7,8,9]. A recent meta-analysis found that the prevalence of PTSD among survivors of infectious diseases after pandemics (i.e., severe acute respiratory syndrome (SARS), H1N1, poliomyelitis, Ebola, Zika, Nipah, Middle Eastern respiratory syndrome coronavirus (MERS-CoV), H5N1, and COVID-19) was 23.8% (95% CI: 16.6 to 31.0) [7]. The development of PTSD for COVID-19 survivors may reflect two mechanisms: (1) the direct experience of severe symptoms and treatments (e.g., dyspnea, respiratory failure, alteration of conscious states, threatening of death, tracheotomy); and (2) fear of re-exposure and social isolation [10]. The temporal process of these two mechanisms may vary across survivors; however, the interconnectedness of PTSD and the biological features of pandemic survivors suggests an increased risk of somatization and biases in pain perception and functional impairment, which ultimately delay rehabilitation outcomes and worsen functioning, well-being, and PTSD symptoms among survivors [6,11,12]. Records of COVID-19 patients with PTSD have shown persistent symptoms even after recovery from the virus [7,13]. This constitutes a public health concern, as this clinical picture perpetuates social challenges, suicidal ideations, and engagement in risky behaviors in this population [14,15].

In general, evidence-based treatments, such as psychotherapy, cognitive-behavioral therapy, and eye movement desensitization and reprocessing, aim to reframe the negative memories associated with traumatic events to diminish negative mood, avoidance behavior, and hyperarousal [16]. It is unclear whether evidence-based psychological interventions will produce similar favorable outcomes for individuals with PTSD triggered by external factors (e.g., combat, abuse) and by somatic factors linked to physical injury or medical trauma (e.g., stroke, cardiac events, cancer) [4,17,18] and who require rehabilitation. A key difference is that, when the cause is external, triggers are centered on past events; in contrast, when the cause is somatic, symptoms are triggered by both past events and the fear of recurrence [4].

Few studies have considered individuals who were diagnosed with PTSD after injury or severe illness and required rehabilitation. COVID-19-specific research is being produced very slowly relative to the number of COVID-19 survivors with disabling sequelae and diagnoses of PTSD [4,10]. The identification of behavioral interventions capable of reducing PTSD symptoms and recovering functioning is critical for bolstering a multidisciplinary rehabilitation approach. This rapid review summarizes the findings on the effectiveness of behavioral interventions intended to improve functioning in adults with PTSD who require rehabilitation services following injury or medical trauma.

## 2. Materials and Methods

This rapid review was informed by the rehabilitation objectives of the World Health Organization (2020). The review adhered to the Preferred Reporting Items for Systematic Reviews and Meta-Analyses (PRISMA) guidelines [19] and the Interim Guidance from the Cochrane Rapid Reviews Methods Group [20] (see Supplementary Table S1, PRISMA 2020 Checklist). The protocol was registered in PROSPERO (CRD42020219933).

### 2.1. Search Strategy and Eligibility Criteria

A search was conducted in PubMed, Embase, and the Cochrane Central Register of Controlled Trials (CENTRAL) databases from inception to 31 March 2021, without language restrictions. The PICO framework was used to develop the search strategy as follows:

*Population:* Adults (aged 18 or over) with a diagnosis of PTSD based on any of the following: DSM-III (APA 1980), DSM-III-R (APA 1987), DSM-IV (APA 2000), DSM-5 (APA 2013), ICD-9 (WHO 1979), or ICD-10 (WHO 1992) were included. The onset and diagnosis of PTSD must have occurred after experiencing physical injury or life-threatening disease (e.g., brain injury, spinal cord injury, neck strain injuries, infectious diseases such as COVID-19), and secondary to comorbidity requiring rehabilitation intervention(s). No restrictions on symptom severity or the type of physical injury or illness (including COVID-19) were applied.
*Interventions:* In the selection of interventions, the authors adhered to the definition that defines rehabilitation as “A multimodal person-centred process, including functioning interventions targeting body functions, and/or activities and participation, and/or the interaction with the environment, with the goal of optimizing functioning for persons with health conditions experiencing disability or likely to experience disability and/or persons with disability” [21]. All behavioral interventions recommended by both the American Psychological Association (APA) and National Institute for Health and Clinical Excellence (NICE) current guidelines for the reduction in PTSD symptoms [3,22] and the improvement of functioning as part of a multidisciplinary rehabilitation treatment were included.
The list of considered interventions comprises the following therapies: cognitive-behavioral therapy, cognitive processing therapy, cognitive therapy, prolonged exposure therapy, brief eclectic psychotherapy, eye movement desensitization and reprocessing therapy, narrative exposure therapy, trauma-focused cognitive-behavioral therapy, supported trauma-focused computerized and cognitive-behavioral therapy—alone or combined with any other treatments and/or usual care. No delivery modality restriction was applied.
*Comparators:* The comparators were PTSD interventions with any other intervention (e.g., usual care, drugs), waitlist, or no-treatment care.
*Outcomes:* Considering the complexity and heterogeneity of the outcomes used in research on PTSD improvement trajectories within the rehabilitative process, the outcomes were categorized and assessed as follows:
*Primary outcomes:*
  - PTSD symptom reduction (e.g., Clinician-Administered PTSD Scale—all versions; PTSD Checklist—all versions);
  - Physical, social, executive, and cognitive functioning (e.g., De Morton Mobility Index, Roland Morris Disability Questionnaire, Global Assessment of Functioning–functioning and symptoms; Wechsler Adult Intelligence Scale—all versions).
*Secondary outcome:*
  - Quality of life (e.g., Human Service Scale Short Form-36 Health Survey; WHO Quality of Life–Brief).

Exclusion criteria comprise the following features:

- Adults diagnosed with PTSD after injury or illness experiencing non-physical (i.e., emotional/psychological) trauma and/or witnessing a traumatic event requiring only behavioral interventions;
- Studies assessing mixed-trauma populations (i.e., with different PTSD-related causes and no separate data), subsyndromal participants (i.e., PTSS), or without an official or clear diagnosis of PTSD, or interventions for the prevention of PTSD;
- Non-RCT study designs such as conference abstracts, protocol stages, pilot, and crossover designs were excluded.

The complete search strategy is displayed in Supplementary Table S2.

### 2.2. Study Selection and Data Extraction

One review author screened titles, abstracts, and full-text articles. Uncertainty was resolved by two other authors. One author extracted data on study characteristics using Microsoft Excel, and any uncertainty regarding study characteristics was addressed and resolved by consultation with a second author. A predetermined data extraction form was used to identify study characteristics; population, intervention, comparison, and outcome (PICO) features; and outcome data of each included paper.

### 2.3. Quality Assessment

Studies that met the selection criteria were independently assessed using the Cochrane risk of bias tool (RoB 1) [23]. The following domains were assessed: random sequence generation, allocation concealment, blinding of participants and personnel, blinding of outcome assessment, incomplete outcome data, selective reporting, and other sources of bias.

Two authors independently assessed each selected study. Any disagreement was resolved by consensus and/or by consultation with a third review author.

### 2.4. Summary of Findings and Assessment of the Certainty of Evidence

The certainty of the body of evidence of the primary outcomes was evaluated using the Grading of Recommendations Assessment, Development, and Evaluation (GRADE) approach [24] and was reported in the Summary of Findings according to Cochrane methodology standards [25]. A single review author applied GRADE, and a second review author verified all judgments and incorporated rationales for judgments in the footnotes.

### 2.5. Data Synthesis

The authors planned to calculate the effect estimate of the interventions by using the risk ratio with a corresponding 95% confidence interval for dichotomous outcomes and by mean difference or standardized mean difference for continuous outcomes and to pool data using a random effect model, as a certain degree of heterogeneity was expected among trials. However, the included studies assessed interventions that were too heterogeneous to make the pooling meaningful. Therefore, a narrative synthesis of the results is presented and reported at the study level.

## 3. Results

**Figure 1:**
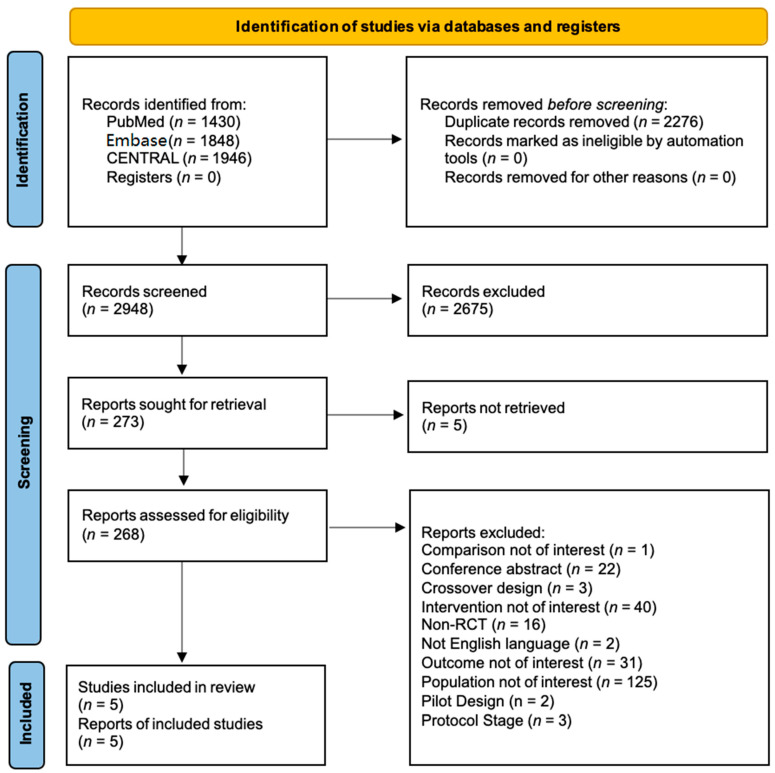
PRISMA-2020 flow diagram.

The search identified 2948 titles and abstracts, excluding duplicates. No additional records were obtained from other sources. Of 268 full-text articles assessed for eligibility, 5 studies met the inclusion criteria to address the study question [26,27,28,29,30], as shown in the PRISMA flow diagram in Figure 1.

After initial screening, 273 reports were retrieved, and 5 articles were not found. After reading the full texts, 263 studies were excluded: of those, 69 reported on individuals with PTSD caused by non-physical trauma/injury; 56 addressed ineligible populations (e.g., subsyndromal, stress, mixed trauma population, unclear/lack of diagnosis); 40 reported on ineligible interventions; 31 reported on ineligible outcomes; 22 were conference abstracts; 19 used ineligible study design (3 crossover design, 16 non-randomized control trials); 19 reported secondary analysis evidence; 3 were protocols; 2 were pilot studies; and finally, 2 full-text manuscripts were not available in English.

### 3.1. Characteristics of Included Studies

The five RCTs [26,27,28,29,30] included 459 adults with PTSD following physical trauma. The sample sizes ranged from 20 to 228, with an average of 92 participants (SD = 75.03). Across the five studies [26,27,28,29,30], three included motor vehicle crash survivors [26,28,30], one included motor vehicle crash survivors with chronic whiplash-associated disorders [29], and one included veterans who sustained a traumatic brain injury [27]. None of the studies included participants with PTSD following COVID-19. All studies followed DSM-IV criteria for PTSD. All studies measured the efficacy of behavioral therapies in reducing PTSD symptoms and were delivered individually. None of the studies assessed group-based interventions. Two studies used a waitlist as a comparator [29,30], one used a different behavioral intervention, one [26] used a pharmacological intervention, and one [28] used a no-treatment control group. Table 1 presents the characteristics of the five included studies. The outcome measures and effect estimates by study are presented in Table 2.

Two studies [29,30] evaluated trauma-focused cognitive-behavioral therapy and cognitive-behavioral therapy against waitlists, respectively. In the remaining three studies [26,27,28], the authors recorded the following comparisons: symptom management and rehabilitation therapy–cognitive processing therapy versus cognitive processing therapy; prolonged exposure therapy versus paroxetine; and cognitive therapy versus repeated assessments. All studies [26,27,28,29,30] reported data on the primary outcome—PTSD symptom severity; two studies measured functioning (disability) [28,29], and one study assessed cognitive function [27]. Finally, two studies [27,29] reported data on quality of life.

### 3.2. Risk of Included Studies

**Figure 2:**
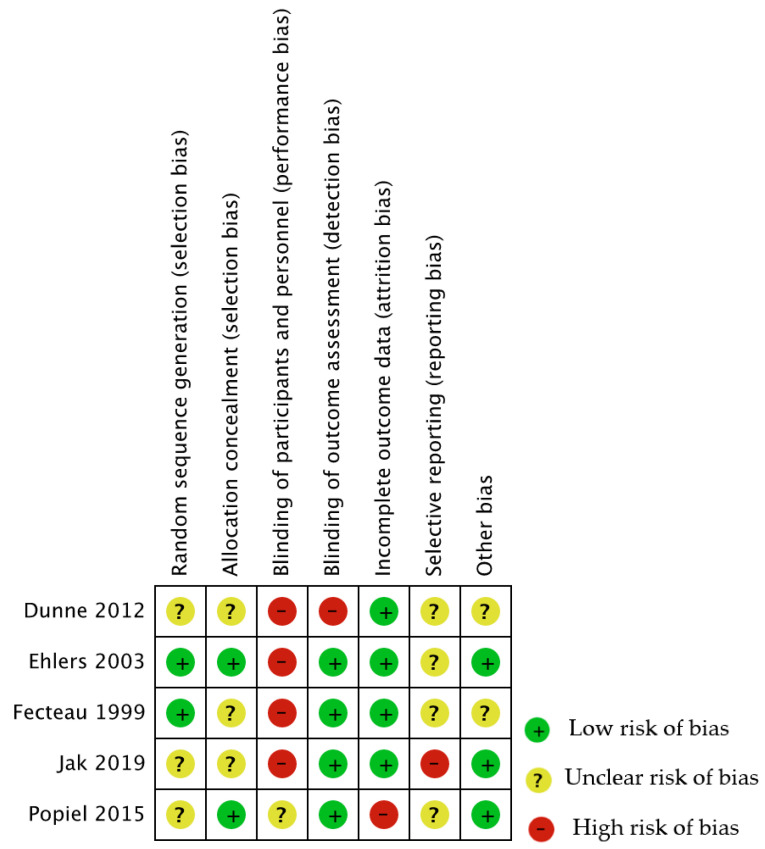
Risk of bias summary [26,27,28,29,30].

**Figure 3:**
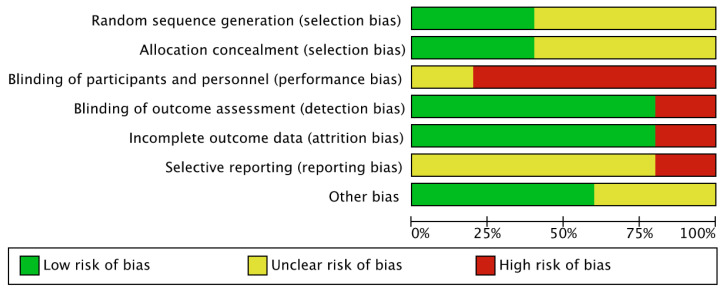
Risk of bias graph.

The risk of bias was generally low or unclear for each domain assessed, except for performance and detection biases, which were judged as high risk in all studies. Figure 2 and Figure 3 provide an overview of the risk of bias for all the considered domains. A detailed description of the assessment is provided in Appendix A.

Five different comparisons of interventions were investigated. The juxtaposition of the five studies revealed high heterogeneity in the interventions and comparators, which prevented a meta-analysis. The report below shows the findings by comparison and describes them at the study level. All details are reported in the online supplemental material.

### 3.3. Effects of Interventions

#### 3.3.1. Cognitive-Behavioral Therapy versus Waitlist

One study [30] compared cognitive-behavioral therapy (*n* = 20) to a waitlist (*n* = 20) among motor vehicle crash survivors and reported data on PTSD symptom reduction measured by the Clinician-Administered PTSD Scale for DSM-5, version 2. The results showed a statistically significant reduction in PTSD symptoms (MD = −37.14; 95% CI = −61.38 to −12.82; very low certainty of evidence).

The outcomes of functioning and quality of life were not assessed.

#### 3.3.2. Trauma-Focused Cognitive-Behavioral Therapy Compared versus Waitlist

One study [29] compared trauma-focused cognitive-behavioral therapy to the waitlist for 23 participants with PTSD in the context of chronic whiplash. There were no statistically significant group differences in physical functioning, as measured by the Neck Disability Index (MD = −5.16; 95% CI = −15.58 to 5.26; very low certainty of the evidence).

When measured with the Post-Traumatic Diagnostic Scale, the trauma-focused cognitive-behavioral therapy group showed a statistically significant decrease in PTSD symptoms, compared with the control group after treatment (MD = −7.69; 95% CI = −14.29 to −1.09; very low certainty of the evidence).

The Short Form 36 Health Survey Questionnaire—36 was employed to assess physical and mental health. The group receiving trauma-focused cognitive-behavioral therapy had higher scores in both domains (MD = 11.00; 95% CI = −2.71 to 24.71; very low certainty of evidence; and MD = 7.31; 95% CI = −8.02 to 22.64; very low certainty of the evidence, respectively), although not statistically significant.

#### 3.3.3. Symptom Management and Rehabilitation Therapy–Cognitive Processing Therapy versus Cognitive Processing Therapy

One study [27] of 100 veterans with traumatic brain injury compared symptom management and rehabilitation therapy–cognitive processing therapy to cognitive processing therapy and assessed cognitive function in six domains: higher level of cognitive processes, psychomotor processing speed, auditory attention and working memory, and verbal learning and recall.

When higher-level cognitive processes were measured with the Wisconsin Card Sorting Test, a statistically significant difference was found in favor of the control group (MD = 6.45; 95% CI = 3 to 9.9). However, no statistically significant difference was observed when higher-level cognitive processes were assessed with the Delis–Kaplan Executive Function System Color–Word Interference and Trail Making Test (MD = −0.28; 95% CI = −2.48 to 1.92; MD = −0.02; 95% CI = −0.8 to 0.76, respectively). The certainty of the evidence was judged to be very low for all outcomes.

The fourth edition of the Wechsler Adult Intelligence Scale was administered to assess psychomotor processing speed (coding and symbol search), and auditory attention and working memory (digit span). No statistically significant difference was found between the two groups for all domains (MD = 0.34; 95% CI = −4.78 to 5.46; and MD = 1.04; 95% CI = −0.16 to 2.24, respectively). The certainty of the evidence was very low for both outcomes.

Learning and recall as measured by the California Verbal Learning Test II were similar in both groups (MD = 4.39; 95% CI = −0.54 to 9.32; very low certainty of the evidence).

PTSD symptom reduction was assessed using the PTSD Checklist—Specific. There were no group differences (MD = −0.48; 95% CI = −6.45 to 5.49; very low certainty of the evidence).

Finally, in measuring the quality of life index with the Quality of Life Inventory-Brief, no effect between groups was found (MD = 0.21; 95% CI = −0.33 to 0.75; very low certainty of the evidence).

#### 3.3.4. Prolonged Exposure Therapy versus Paroxetine

One study [26] compared prolonged exposure therapy to paroxetine and reported a reduction in PTSD symptoms in 140 adults who experienced a motor vehicle crash. Assessed using the Post-Traumatic Diagnostic Scale, the paroxetine group reported a non-significantly greater rate of symptom reduction than the prolonged exposure therapy group (RR = 1.51; 95% CI = 0.98 to 2.32; very low certainty of the evidence).

Functioning and quality of life were not examined in this study.

#### 3.3.5. Cognitive Therapy versus Repeated Assessments

One study [28] compared cognitive therapy with repeated assessments in 79 participants with PTSD following a motor vehicle crash. When examining functioning (disability) with Sheehan Disability Scale, participants who received the intervention performed marginally better than those in the control group (MD = −1.90; 95% CI =−3.17 to −0.63; very low certainty of the evidence).

The group receiving cognitive therapy reported a significantly lower PTSD symptom frequency than the repeated assessments group (Post-Traumatic Stress Diagnostic Scale: MD = −14.30; 95% CI = −20.05 to −8.55; Clinician-Administered PTSD Scale -SX: MD = −14.40; 95% CI = −20.66 to −8.14). Similarly, PTSD symptom intensity (Clinician-Administered PTSD Scale -SX: MD = −12.20; 95% CI = −17.95 to −6.45) and distress (Post-Traumatic Stress Diagnostic Scale: MD −12.50; 95% CI = −18.55 to −6.45) also favored the cognitive therapy group, with statistically significant results. The certainty of the evidence was low for all outcomes.

Quality of life was not an outcome in this RCT.

### 3.4. Certainty of Evidence

The certainty of the evidence of review outcomes was very low across all studies; it was downgraded for risk of bias due to the high risk of performance and detection bias and for the imprecision of the estimates due to the small number of studies and participants (Supplementary Tables S3–S7). These methodological limitations can lead to an overestimation of the effects of the intervention [31].

## 4. Discussion

This rapid review examined the efficacy of behavioral interventions in decreasing PTSD symptom severity, reducing patients’ perceived level of disability, and improving cognitive function and quality of life among individuals who sustained physical trauma (i.e., chronic whiplash-associated disorders, motor vehicle accident survivors, traumatic brain injury) and required rehabilitation services. No RCTs assessing ICU admission, pandemic, and cancer survivors met the inclusion criteria. These RCTs either provided preliminary data or focused on clinicians, caregivers, PTSS, or PTSD preventive interventions. Among the PTSD treatment options recommended by the APA and NICE, the efficacy of five interventions was assessed: trauma-focused cognitive-behavioral therapy, cognitive-behavioral therapy, cognitive processing therapy, prolonged exposure therapy, and cognitive therapy.

A recent systematic review found that trauma-focused therapies are more effective than pharmacological interventions in treating PTSD [32]. This result is supported by other researchers who detected greater improvements among individuals treated with a psychotherapeutic approach than among those treated with a pharmacological regimen [32,33]. However, the review of the evidence reported in the five RCTs is insufficient to ascertain any advantage or disadvantage of these interventions in patients with PTSD following physical trauma or illness. This uncertainty aligns with the suggestion that the diverse causes of PTSD identified in individuals who have experienced physical trauma or illness may require updated measurement scales and interventions that differ from those used when PTSD is triggered by psychological trauma. Individuals who develop PTSD after a somatic event require broader and more complex medical attention than those provided by mental health providers [4,34]. These might include COVID-19 survivors with PTSD whose sense of threat has been linked to somatic issues [35]. Therefore, to achieve the goal of optimizing and tailoring multidisciplinary rehabilitative services to this population, increased efforts are needed to better understand this phenomenon and the variation in outcomes among individuals who have sustained a physical injury or have experienced a life-threatening medical event.

### 4.1. PTSD in COVID-19 Survivors

Severely ill COVID-19 patients often remain on a ventilator for a longer period than typical intensive care treatment, which can lead to PTSD symptoms among survivors [8,36]. This population is also likely to require rehabilitation services owing to a variety of challenges with normal post-recovery functioning [9,13,37]. However, PTSD can hinder recovery and rehabilitation outcomes, as the perception of pain and illness is amplified in these patients. Further efforts are needed to explore the efficacy of behavioral interventions for PTSD symptoms among patients who require rehabilitation services by recruiting larger samples and by employing rigorous methodologies that constrain the risk of bias to bolster the quality of the evidence and generalizability to the rehabilitation and COVID-19 survivor populations.

It is also critical to note that delayed onset of PTSD occurs in approximately 25% of cases, which may explain how the estimate of COVID-19 survivors with PTSD reached 30% [13,38]. Continued screening of COVID-19 survivors after recovery with reliable, valid, and sensitive tools and screening for risk factors on a case-by-case basis may prevent the onset of PTSD and ensure the prompt delivery of services to minimize the risk of negative outcomes [13]. As part of multidisciplinary and interdisciplinary rehabilitation services, better outcomes are observed when the focus is on physical and psychological treatments, and accessibility to service is timely.

### 4.2. Limitations and Implications

Considering the condensed timeframe that characterizes a rapid review, studies not published in English could not be considered for inclusion. The findings are reflective of five countries, which may limit its global applicability. The authors are confident to have included all relevant studies based on review questions, inclusion, and exclusion criteria; however, databases were last searched on 31 March 2021, and newly published RCTs may be missing.

Despite these limitations, this review brings attention to an important topic for the rehabilitation and mental health communities: PTSD triggered by medical trauma. Determining the temporal antecedents from onset of first symptoms to diagnosis is not linear, and isolating the trigger(s) may be impossible when there are pre-existing conditions, both diagnosed and undiagnosed. This review highlights that the mechanisms triggering PTSD should be understood across a wide spectrum of possibilities and not restricted to psychological trauma with little attention paid to the cause.

This line of reasoning has already been introduced in ICU studies: “whilst life-threatening injuries requiring ICU admission are frequently perceived as more psychologically traumatic, there is an increasing body of evidence suggesting a dependent relationship between ICU admission and the later development of PTSD, irrespective of the events preceding ICU admission” [39]. The authors thereby invite the scientific community to:

1. Focus investigations on the etiology and mechanisms by which trauma causes PTSD;
2. Provide clear definition;
3. Spread awareness on critical differences that may impact clinical outcomes among patients who survived a life-threatening disease and those who experience psychological trauma.

Sedation vacation is often used among ICU patients to prevent delirium, and it may reduce the risk of developing PTSD. However, sedation vacation may not be possible in patients with severe acute respiratory distress. COVID-19 patients and survivors face limited preventive and treatment options for PTSD, partially explaining the continued increase in diagnosis. Further studies shall investigate preventive and treatment options for people who experienced life-threatening diseases leading to an ICU stay. However, the population of interest, and in this case, the condition (PTSS vs. PTSD), need to be identified to develop a clear research question. This approach will help select the proper study design and methodology, leading to a higher quality of evidence [40]. Finally, in examining the role of rehabilitation psychology in the context of clinical rehabilitation, psychological interventions shall not be compartmentalized but instead measured as part of the multidisciplinary rehabilitation program that supports recovery of functioning.

## 5. Conclusions

Although behavioral interventions, such as cognitive therapy, cognitive-behavioral therapy, trauma-focused cognitive-behavioral therapy, cognitive processing therapy, and prolonged exposure therapy, are effective in reducing PTSD symptoms and improving function and quality of life among adults who receive a diagnosis of PTSD after experiencing psychological trauma [32,33], it is uncertain whether patients diagnosed with PTSD following physical trauma, such as illness or injury, who require subsequent rehabilitation services, achieve similar clinical improvements.

## Figures and Tables

**Table 1:** Characteristics of included studies.

Author(s) (Year)	Title	Country	Aims	Type of Population	No. PP	Diagnostic Instruments
Dunne et al. (2012) [29]	A Randomized Controlled Trial of Cognitive-Behavioral Therapy for the Treatment of PTSD in the Context of Chronic Whiplash.	Australia	Assess if trauma-focused cognitive-behavioral therapy reduces PTSD diagnosis, symptoms, and physiological reactivity to trauma cues.	Chronic whiplash-associated disorders	26	Structured clinical interview for DSM-IV
Fecteau and Nicki (1999) [30]	Cognitive-Behavioural Treatment of Post-Traumatic Stress Disorder after Motor Vehicle Accident.	Canada	Evaluate the efficacy of cognitive-behavioral therapy for PTSD following motor vehicle accident trauma.	Motor vehicle accident survivors	20	Clinically administrated PTSD scale-2 for DSM-IV
Jak et al. (2019) [27]	SMART-CPT for Veterans with Comorbid Post-Traumatic Stress Disorder and History of Traumatic Brain Injury: A Randomised Controlled Trial.	USA	Compare a novel treatment, symptom management, and rehabilitation therapy—cognitive processing therapy to cognitive processing therapy to evaluate its efficacy in PTSD symptoms and cognitive function improvements.	Veterans with traumatic brain injury	100	Clinically administrated PTSD scale-2 DSM-IV
Popiel et al. (2015) [26]	Prolonged Exposure, Paroxetine and the Combination in the Treatment of PTSD Following a Motor Vehicle Accident. A Randomized Clinical trial—The “TRAKT” study.	Poland	Compared the efficacy of prolonged exposure and medication when used as a monotherapy in treating PTSD.	Motor vehicle accident survivors	228	Structured clinical interview for DSM-IV
Ehlers et al. (2003) [28]	A Randomized Controlled Trial of Cognitive Therapy, a Self-Help Booklet, and Repeated Assessments AS Early Interventions for Posttraumatic Stress Disorder.	UK	Determine whether cognitive therapy or a self-help booklet is more effective in preventing chronic PTSD than repeated assessments.	Motor vehicle accident survivors	85	Structured clinical interview for DSM-IV; Post-traumatic diagnostic scale

Abbreviations: PTSD = post-traumatic stress disorder; DSM-IV = Diagnostic and Statistical Manual of Mental Disorders IV edition.

**Table 2:** Outcomes and effect estimates of included studies.

Author(s) (Year)	Comparison	Outcomes	Outcome Measures	PP Analyzed	Effect Estimates (95% CI)
Dunne et al. (2012) [29]	Trauma-focused cognitive-behavioral therapy vs. Waitlist	PTSD Symptoms Severity	Post-Traumatic Diagnostic Scale	23	−7.69 (−14.29–−1.09)
		Quality of Life	Short Form Health Survey-36 (Physical Health Total)		11.00 (−2.71–24.71)
			Short Form Health Survey-36 (Mental Health Total)		7.31 (−8.02–22.64)
		Disability	Neck Disability Index		−5.16 (−15.58–5.26)
Fecteau and Nicki (1999) [30]	Cognitive-behavioral therapy vs. waitlist	PTSD Symptoms Severity	Clinically Administrated PTSD Scale-2	20	−37.10 (−61.38–−12.82)
Jak et al. (2019) [27]	Symptom management and rehabilitation therapy–cognitive processing therapy vs. Cognitive processing therapy	PTSD Symptoms Severity	PTSD Check List-Specific	100	−0.48 (−6.45–5.49)
		Cognitive Function	Wechsler Adult Intelligence Scale-IV (Processing Speed)		0.34 (−4.78–5.46)
			Wechsler Adult Intelligence Scale-IV (Digit Span)		1.04 (−2.48–1.92)
			Delis–Kaplan Executive Function System (Color–Word Interference)		−0.28 (−0.16–2.24)
			Delis–Kaplan Executive Function System (Trail Making)		−0.02 (−0.80–0.76)
			Wisconsin Card Sorting Test—64 (Total Errors)		6.45 (3.00–9.90)
			California Verbal Learning Test II (Learning Tot.)		4.39 (−0.54–9.32)
		General Life Satisfaction	Quality of Life Inventory-B		0.21 (−0.33–0.75)
Popiel et al. (2015) [26]	Prolonged exposure vs. paroxetine	Symptoms Remission	Post-traumatic Diagnostic Scale	140	1.51 (−0.98–2.32)
Ehlers et al. (2003) [28]	Cognitive therapy vs. repeated assessments	PTSD Symptoms Severity	Post-Traumatic Diagnostic Scale (Frequency)	54	−14.30 (−20.05–−8.55)
			Post-Traumatic Diagnostic Scale (Distress)		−12.50 (−18.55–−6.45)
			Clinically Administrated PTSD Scale-SX (Frequency)		−14.40 (−20.66–−8.14)
			Clinically Administrated PTSD Scale-SX (Intensity)		−14.40 (−20.66–−8.14)
		Disability	Sheehan Disability Scale		−1.90 (−3.17–−0.63)
			Clinically Administrated PTSD Scale-SX (Disability)		−0.70 (−1.10–−0.30)

Abbreviations: PTSD = post-traumatic stress disorder; CI = confidence interval.

## Data Availability

Not applicable.

## Supplementary Materials

The following supporting information can be downloaded at: https://www.mdpi.com/article/10.3390/ijerph19127514/s1. Supplementary Table S1. PRISMA 2020 Checklist. Supplementary Table S2. Search Strategy. Supplementary Table S3. Cognitive behavioral therapy compared to waiting list for PTSD. Supplementary Table S4. Trauma-focused cognitive behavioral therapy compared to waiting list for PTSD. Supplementary Table S5. Symptom management and rehabilitation therapy-cognitive processing therapy compared to cognitive processing therapy for PTSD. Supplementary Table S6. Prolonged exposure therapy compared to paroxetine for PTSD. Supplementary Table S7. Cognitive therapy compared to repeated assessments for PTSD. Reference [19] is cited in the supplementary materials.

## Appendix A. Risk of Bias

- **Random Sequence Generation (selection bias)**

Three studies [27,28,30] did not provide enough information about the sequence generation process. The other two studies [29,31] described an appropriate method of sequence generation and were judged to be at low risk of bias.

- **Allocation Concealment (selection bias)**

Three studies [28,30,31] did not provide sufficient information on concealment methods to permit the assessment of whether the allocation sequence was concealed. Two studies [27,29] reported the concealment of allocation and were judged to be at low risk of selection bias.

- **Blinding of Participants and Personnel (performance bias)**

Blinding of participants and providers was not achievable due to the nature of the psychological interventions delivered during the RCTs. Thus, the risk of bias was rated high in all studies [27,28,29,30,31].

- **Blinding of Outcome Assessor (detection bias)**

Blinding of outcome assessors (the participants) was not attainable in any of the five RCTs [27,28,29,30,31]. Reasons for this rating include the use of self-reporting instruments to measure the primary outcomes [27,28], the comparison of the intervention group against a waitlist [30,31], no intervention (repeated assessments) [29], or conventional therapy [28] group. Hence, all studies were judged as high risk of bias.

- **Incomplete Data Outcome (attrition bias)**

One study [27] was judged as high in risk of attrition bias because of an unbalanced drop-out rate between groups. All other studies [28,29,30,31] experienced little or no dropout rates and were considered at low risk of bias.

- **Selective Reporting**

The limited information provided in four studies [27,29,30,31], together with the inability to locate study protocols, made it difficult to draw definite conclusions in this domain. We thereby judged these studies unclear in the risk of selective reporting bias. One study [28] was judged to be high in risk because of observed discrepancies between protocol and manuscript.
