# Supplementary material for: The Effectiveness of Behavioral Interventions in Adults with Post-Traumatic Stress Disorder during Clinical Rehabilitation: A Rapid Review
Source: Int J Environ Res Public Health. 2022 Jun 19;19(12):7514. doi: 10.3390/ijerph19127514 (PMC9224304; doi:10.3390/ijerph19127514)
Supplement: Supplementary file 1 [file ijerph-19-07514-s001.zip › ijerph-1763555-supplementary.pdf]

Supplementary Table S1. PRISMA 2020 Checklist.

Section and Topic	Item #	Checklist Item	Location Where Item Is Reported
**TITLE**			
Title	1	Identify the report as a systematic review.	1
**ABSTRACT**			
Abstract	2	See the PRISMA 2020 for Abstracts checklist.	2
**INTRODUCTION**			
Rationale	3	Describe the rationale for the review in the context of existing knowledge.	4,5
Objectives	4	Provide an explicit statement of the objective(s) or question(s) the review addresses.	5
**METHODS**			
Eligibility criteria	5	Specify the inclusion and exclusion criteria for the review and how studies were grouped for the syntheses.	6,7
Information sources	6	Specify all databases, registers, websites, organisations, reference lists and other sources searched or consulted to identify studies. Specify the date when each source was last searched or consulted.	6
Search strategy	7	Present the full search strategies for all databases, registers and websites, including any filters and limits used.	Supplementary Table S2
Selection process	8	Specify the methods used to decide whether a study met the inclusion criteria of the review, including how many reviewers screened each record and each report retrieved, whether they worked independently, and if applicable, details of automation tools used in the process.	7,8
Data collection process	9	Specify the methods used to collect data from reports, including how many reviewers collected data from each report, whether they worked independently, any processes for obtaining or confirming data from study investigators, and if applicable, details of automation tools used in the process.	7,8
Data items	10a	List and define all outcomes for which data were sought. Specify whether all results that were compatible with each outcome domain in each study were sought (e.g. for all measures, time points, analyses), and if not, the methods used to decide which results to collect.	7
	10b	List and define all other variables for which data were sought (e.g. participant and intervention characteristics, funding sources). Describe any assumptions made about any missing or unclear information.	6,7
Study risk of bias assessment	11	Specify the methods used to assess risk of bias in the included studies, including details of the tool(s) used, how many reviewers assessed each study and whether they worked independently, and if applicable, details of automation tools used in the process.	8
Effect measures	12	Specify for each outcome the effect measure(s) (e.g. risk ratio, mean difference) used in the synthesis or presentation of results.	8
Synthesis methods	13a	Describe the processes used to decide which studies were eligible for each synthesis (e.g. tabulating the study intervention characteristics and comparing against the planned groups for each synthesis (item #5)).	7,8
	13b	Describe any methods required to prepare the data for presentation or synthesis, such as handling of missing summary statistics, or data conversions.	7,8
	13c	Describe any methods used to tabulate or visually display results of individual studies and syntheses.	7,8
	13d	Describe any methods used to synthesize results and provide a rationale for the choice(s). If meta-analysis was performed, describe the model(s), method(s) to identify the presence and extent of statistical heterogeneity, and software package(s) used.	7,8
	13e	Describe any methods used to explore possible causes of heterogeneity among study results (e.g. subgroup analysis, meta-	7,9

Section and Topic	Item #	Checklist Item	Location Where Item Is Reported
		regression).	
	13f	Describe any sensitivity analyses conducted to assess robustness of the synthesized results.	10,11
Reporting bias assessment	14	Describe any methods used to assess risk of bias due to missing results in a synthesis (arising from reporting biases).	8
Certainty assessment	15	Describe any methods used to assess certainty (or confidence) in the body of evidence for an outcome.	8
**RESULTS**			
Study selection	16a	Describe the results of the search and selection process, from the number of records identified in the search to the number of studies included in the review, ideally using a flow diagram.	9, Figure 1
	16b	Cite studies that might appear to meet the inclusion criteria, but which were excluded, and explain why they were excluded.	9, eTable1
Study characteristics	17	Cite each included study and present its characteristics.	9, Table 1
Risk of bias in studies	18	Present assessments of risk of bias for each included study.	10, Supplementary Table S3
Results of individual studies	19	For all outcomes, present, for each study: (a) summary statistics for each group (where appropriate) and (b) an effect estimate and its precision (e.g. confidence/credible interval), ideally using structured tables or plots.	10-13
Results of syntheses	20a	For each synthesis, briefly summarise the characteristics and risk of bias among contributing studies.	10-13
	20b	Present results of all statistical syntheses conducted. If meta-analysis was done, present for each the summary estimate and its precision (e.g. confidence/credible interval) and measures of statistical heterogeneity. If comparing groups, describe the direction of the effect.	N/A
	20c	Present results of all investigations of possible causes of heterogeneity among study results.	10
	20d	Present results of all sensitivity analyses conducted to assess the robustness of the synthesized results.	10-13
Reporting biases	21	Present assessments of risk of bias due to missing results (arising from reporting biases) for each synthesis assessed.	10
Certainty of evidence	22	Present assessments of certainty (or confidence) in the body of evidence for each outcome assessed.	13, eTables 2-6
**DISCUSSION**			
Discussion	23a	Provide a general interpretation of the results in the context of other evidence.	13,14
	23b	Discuss any limitations of the evidence included in the review.	13,14
	23c	Discuss any limitations of the review processes used.	13
	23d	Discuss implications of the results for practice, policy, and future research.	15
**OTHER INFORMATION**			
Registration and protocol	24a	Provide registration information for the review, including register name and registration number, or state that the review was not registered.	5
	24b	Indicate where the review protocol can be accessed, or state that a protocol was not prepared.	5
	24c	Describe and explain any amendments to information provided at registration or in the protocol.	5

Section and Topic	Item #	Checklist Item	Location Where Item Is Reported
Support	25	Describe sources of financial or non-financial support for the review, and the role of the funders or sponsors in the review.	1
Competing interests	26	Declare any competing interests of review authors.	1
Availability of data, code and other materials	27	Report which of the following are publicly available and where they can be found: template data collection forms; data extracted from included studies; data used for all analyses; analytic code; any other materials used in the review.	/

From: Page MJ, McKenzie JE, Bossuyt PM, Boutron I, Hoffmann TC, Mulrow CD, et al. The PRISMA 2020 statement: an updated guideline for reporting systematic reviews. BMJ 2021;372:n71. doi: 10.1136/bmj.n71

For more information, visit: http://www.prisma-statement.org/

Supplementary Table S2. Search Strategy.

Database	Search Strategy
**PubMed (via pubmed.ncbi.nlm.nih.gov/)**	1. "Stress Disorders, Post-Traumatic"[Mesh]<br>2. PTSD[tiab] OR ((posttrauma*[tiab] OR post-trauma*[tiab] OR "post trauma*"[tiab]) AND (stress*[tiab] OR disorder*[tiab] OR psych*[tiab] OR symptom*[tiab])) OR "combat disorder*"[tiab] OR "war neuros*"[tiab]<br>3. #1 OR #2<br>4. "Cognitive Behavioral Therapy"[Mesh]<br>5. "Cognitive Behavioral Therap*"[tiab] OR "Cognitive Behavior Therap*"[tiab] OR "Cognitive Behavioural Therap*"[tiab] OR "Cognitive Behaviour Therap*"[tiab] OR CBT[tiab]<br>6. "Eye Movement Desensitization Reprocessing"[Mesh]<br>7. "Eye Movement Desensitization and Processing"[tiab] OR "Eye Movement Desensitization and reprocessing"[tiab] OR "Eye Movement Desensitization reprocessing"[tiab] OR EMDR[tiab]<br>8. "Psychotherapy, Brief"[Mesh]<br>9. "Brief Psychotherap*"[tiab] OR "Brief Eclectic Psychotherap*"[tiab] OR "short-term psychotherap*"[tiab] OR "Solution-Focused Brief Therap*"[tiab]<br>10. "cognitive processing therap*"[tiab] OR CPT[tiab]<br>11. "cognitive therap*"[tiab] OR "Cognition therap*"[tiab] OR "Cognitive Psychotherap*"[tiab]<br>12. "exposure-based therap*"[tiab] OR "Prolonged exposure therap*"[tiab]<br>13. "Narrative Therapy"[Mesh]<br>14. "Narrative Exposure Therap*"[tiab] OR NET[tiab]<br>15. #4 OR #5 OR #6 OR #7 OR #8 OR #9 OR #10 OR #11 OR #12 OR #13 OR #14<br>16. "randomized controlled trial"[pt]<br>17. "controlled clinical trial"[pt]<br>18. randomized[tiab]<br>19. placebo[tiab]<br>20. "clinical trials as topic"[mesh: noexp]<br>21. "randomly"[tiab]<br>22. "trial"[ti]<br>23. #16 OR #17 OR #18 OR #19 OR #20 OR #21 OR #22<br>24. animals [mh] NOT humans [mh]<br>25. #23 NOT #24<br>26. #3 AND #15 AND #25
**Embase (via Embase.com)**	1. 'posttraumatic stress disorder'/exp<br>2. (PTSD OR ((posttrauma* OR post-trauma* OR 'post trauma*') NEAR/3 (stress* OR disorder* OR psych* OR symptom*)) OR 'combat disorder*' OR 'war neuros*'):ti,ab,kw<br>3. #1 OR #2<br>4. 'cognitive behavioral therapy'/exp<br>5. ('cognitive behavio$ral therap*' OR 'cognitive behavio$r therap*' OR CBT):ti,ab,kw<br>6. 'cognitive therapy'/exp<br>7. ((cognitive OR cognition) NEXT/1 (therap* OR Psychotherap*)):ti,ab,kw

8. 'cognitive processing therapy'/exp
9. ('cognitive processing therap*' OR CPT):ti,ab,kw
10. 'eye movement desensitization and reprocessing'/exp
11. ('Eye Movement Desensitization and Processing' OR 'Eye Movement Desensitization and reprocessing' OR 'Eye Movement Desensitization reprocessing' OR EMDR):ti,ab,kw
12. 'short term psychotherapy'/exp
13. ('Brief Psychotherap*' OR 'Brief Eclectic Psychotherap*' OR 'short-term psychotherap*' OR 'Solution-Focused Brief Therap*'):ti,ab,kw
14. 'prolonged exposure therapy'/exp
15. ('exposure-based therap*' OR 'Prolonged exposure therap*'):ti,ab,kw
16. 'narrative exposure therapy'/exp
17. ('Narrative Exposure Therap*' OR NET):ti,ab,kw
18. #4 OR #5 OR #6 OR #7 OR #8 OR #9 OR #10 OR #11 OR #12 OR #13 OR #14 OR #15 OR #16 OR #17
19. 'randomized controlled trial'/de
20. 'controlled clinical trial'/de
21. random*:ti,ab,tt
22. 'randomization'/de
23. 'intermethod comparison'/de
24. placebo:ti,ab,tt
25. (compare:ti,tt OR compared:ti,tt OR comparison:ti,tt)
26. ((evaluated:ab OR evaluate:ab OR evaluating:ab OR assessed:ab OR assess:ab) AND (compare:ab OR compared:ab OR comparing:ab OR comparison:ab))
27. (open NEXT/1 label):ti,ab,tt
28. ((double OR single OR doubly OR singly) NEXT/1 (blind OR blinded OR blindly)):ti,ab,tt
29. 'double blind procedure'/de
30. (parallel NEXT/1 group*):ti,ab,tt
31. (crossover:ti,ab,tt OR 'cross over':ti,ab,tt)
32. ((assign* OR match OR matched OR allocation) NEAR/6 (alternate OR group OR groups OR intervention OR interventions OR patient OR patients OR subject OR subjects OR participant OR participants)):ti,ab,tt
33. (assigned:ti,ab,tt OR allocated:ti,ab,tt)
34. (controlled NEAR/8 (study OR design OR trial)):ti,ab,tt
35. (volunteer:ti,ab,tt OR volunteers:ti,ab,tt)
36. 'human experiment'/de
37. Trial:ti,tt
38. #19 OR #20 OR #21 OR #22 OR #23 OR #24 OR #25 OR #26 OR #27 OR #28 OR #29 OR #30 OR #31 OR #32 OR #33 OR #34 OR #35 OR #36 OR #37
39. (((random* NEXT/1 sampl* NEAR/8 ('cross section*' OR questionnaire* OR survey OR surveys OR database or databases)):ti,ab,tt) NOT ('comparative study'/de OR 'controlled

	study'/de OR 'randomised controlled':ti,ab,tt OR 'randomized controlled':ti,ab,tt OR 'randomly assigned':ti,ab,tt))<br>40. ('cross-sectional study'/de NOT ('randomized controlled trial'/de OR 'controlled clinical study'/de OR 'controlled study'/de OR 'randomised controlled':ti,ab,tt OR 'randomized controlled':ti,ab,tt OR 'control group':ti,ab,tt OR 'control groups':ti,ab,tt))<br>41. ('case control*':ti,ab,tt AND random*:ti,ab,tt NOT ('randomised controlled':ti,ab,tt OR 'randomized controlled':ti,ab,tt))<br>42. ('systematic review':ti,tt NOT (trial:ti,tt OR study:ti,tt))<br>43. (nonrandom*:ti,ab,tt NOT random*:ti,ab,tt)<br>44. 'random field*':ti,ab,tt<br>45. ('random cluster' NEAR/4 sampl*):ti,ab,tt<br>46. (review:ab AND review:it NOT trial:ti,tt)<br>47. ('we searched':ab AND (review:ti,tt OR review:it))<br>48. 'update review':ab<br>49. (databases NEAR/5 searched):ab<br>50. ((rat:ti,tt OR rats:ti,tt OR mouse:ti,tt OR mice:ti,tt OR swine:ti,tt OR porcine:ti,tt OR murine:ti,tt OR sheep:ti,tt OR lambs:ti,tt OR pigs:ti,tt OR piglets:ti,tt OR rabbit:ti,tt OR rabbits:ti,tt OR cat:ti,tt OR cats:ti,tt OR dog:ti,tt OR dogs:ti,tt OR cattle:ti,tt OR bovine:ti,tt OR monkey:ti,tt OR monkeys:ti,tt OR trout:ti,tt OR marmoset*:ti,tt) AND 'animal experiment'/de)<br>51. ('animal experiment'/de NOT ('human experiment'/de OR 'human'/de))<br>52. #39 OR #40 OR #41 OR #42 OR #43 OR #44 OR #45 OR #46 OR #47 OR #48 OR #49 OR #50 OR #51<br>53. #38 NOT #52<br>54. #3 AND #18 AND #53
**CENTRAL (via Cochrane Library)**	1. MeSH descriptor: [Stress Disorders, Post-Traumatic] explode all trees<br>2. (PTSD OR ((posttrauma* OR post-trauma* OR (post NEXT trauma*)) NEAR/3 (stress* OR disorder* OR psych* OR symptom*)) OR (combat NEXT disorder*) OR (war NEXT neuros*)):ti,ab,kw<br>3. #1 OR #2<br>4. MeSH descriptor: [Cognitive Behavioral Therapy] explode all trees<br>5. ((("Cognitive Behavioral" OR "Cognitive Behavior" OR "Cognitive Behavioural" OR "Cognitive Behaviour") NEXT Therap*) OR CBT):ti,ab,kw<br>6. MeSH descriptor: [Eye Movement Desensitization Reprocessing] explode all trees<br>7. ("Eye Movement Desensitization and Processing" OR "Eye Movement Desensitization and reprocessing" OR "Eye Movement Desensitization reprocessing" OR EMDR):ti,ab,kw<br>8. ((cognitive OR cognition) NEXT (therap* OR Psychotherap*)):ti,ab,kw<br>9. MeSH descriptor: [Psychotherapy, Brief] explode all trees<br>10. (((Brief OR "Brief Eclectic" OR "short term") NEXT psychotherap*) OR ("Solution focused brief" NEXT Therap*)):ti,ab,kw<br>11. (("cognitive processing" NEXT therap*) OR CPT):ti,ab,kw<br>12. (("exposure based" OR "Prolonged exposure") NEXT therap*):ti,ab,kw

13. MeSH descriptor: [Narrative Therapy] explode all trees
14. (("Narrative Exposure" NEXT Therap*) OR NET):ti,ab,kw
15. #4 OR #5 OR #6 OR #7 OR #8 OR #9 OR #10 OR #11 OR #12 OR #13 OR #14
16. #3 AND #15 – in Trials

Supplementary Table S3. Cognitive behavioral therapy compared to waiting list for PTSD.

Certainty assessment							№ of patients		Effect		Certainty	Importance
№ of Studies	Study Design	Risk of Bias	Inconsistency	Indirectness	Imprecision	Other Considerations	CBT	WL	Relative (95% CI)	Absolute (95% CI)		
**PTSD Symptoms Severity (CAPS-2) - Overall**												
1	randomised trials	serious [a]	not serious	not serious	very serious [b]	none	10	10	-	MD **37.1 lower** (61.38 lower to 12.82 lower)	⨁◯◯◯ VERY LOW	

CI: Confidence interval; **MD:** Mean difference

Explanations

a. performance bias, detection bias

b. sample size <300 patients

Supplementary Table S4. Trauma-focused cognitive behavioral therapy compared to waiting list for PTSD.

Certainty assessment							№ of patients		Effect		Certainty	Importance
№ of Studies	Study Design	Risk of Bias	Inconsistency	Indirectness	Imprecision	Other Considerations	TF-CBT	WL	Relative (95% CI)	Absolute (95% CI)		
**PTSD Symptoms Severity - PDS**												
1	randomised trials	serious [a]	not serious	not serious	very serious [b]	none	12	11	-	MD **7.69 lower** (14.29 lower to 1.09 lower)	⨁◯◯◯ VERY LOW	
**Disability NDI**												
1	randomised trials	serious [a]	not serious	not serious	very serious [b]	none	12	11	-	MD **5.16 lower** (15.58 lower to 5.26 higher)	⨁◯◯◯ VERY LOW	
**Quality of Life (SF-36) - Physical Health Tot**												
1	randomised trials	serious [a]	not serious	not serious	very serious [b]	none	12	11	-	MD **11 higher** (2.71 lower to 24.71 higher)	⨁◯◯◯ VERY LOW	
**Quality of Life (SF-36) - Mental Health Tot**												
1	randomised trials	serious [a]	not serious	not serious	very serious [b]	none	12	11	-	MD **7.31 higher** (8.02 lower to 22.64 higher)	⨁◯◯◯ VERY LOW	

CI: Confidence interval; **MD:** Mean difference

Explanations

a. performance and detection bias

b. sample size <300 patients

Supplementary Table S5. Symptom management and rehabilitation therapy-cognitive processing therapy compared to cognitive processing therapy for PTSD.

Certainty Assessment							№ of Patients		Effect		Certainty	Importance
№ of Studies	Study Design	Risk of Bias	Inconsistency	Indirectness	Imprecision	Other Considerations	SMART-CPT	CPT	Relative (95% CI)	Absolute (95% CI)		
**PTSD Symptoms Severity (PCL-S)**												
1	randomised trials	serious [a]	not serious	not serious	very serious [b]	none	51	49	-	MD **0.48 lower** (6.45 lower to 5.49 higher)	⊕◯◯◯ VERY LOW	
**Cognition (WAIS-IV) Processing Speed Index**												
1	randomised trials	serious [a]	not serious	not serious	very serious [b]	none	51	49	-	MD **0.34 higher** (4.78 lower to 5.46 higher)	⊕◯◯◯ VERY LOW	
**Cognition (WAIS-IV) Digit Span**												
1	randomised trials	serious [a]	not serious	not serious	very serious [b]	none	51	49	-	MD **1.04 higher** (0.16 lower to 2.24 higher)	⊕◯◯◯ VERY LOW	
**Cognition (CVLT-II) Learning Tot**												
1	randomised trials	serious [a]	not serious	not serious	very serious [b]	none	51	49	-	MD **4.39 higher** (0.54 lower to 9.32 higher)	⊕◯◯◯ VERY LOW	
**Higher-level Cognitive Processes (WCST) Total Errors**												
1	randomised trials	serious [a]	not serious	not serious	very serious [b]	none	51	49	-	MD **6.45 higher** (3 higher to 9.9 higher)	⊕◯◯◯ VERY LOW	
**Higher-level Cognitive Processes (D-KEFS Trail Making Switching)**												
1	randomised trials	serious [a]	not serious	not serious	very serious [b]	none	51	49	-	MD **0.02 lower** (0.8 lower to 0.76 higher)	⊕◯◯◯ VERY LOW	
**Higher-level Cognitive Processes (D-KEFS CWI)**												
1	randomised trials	serious [a]	not serious	not serious	very serious [b]	none	51	49	-	MD **0.28 lower** (2.48 lower to 1.92 higher)	⊕◯◯◯ VERY LOW	
**General Life Satisfaction (QOLI-B)**												

Certainty Assessment							№ of Patients		Effect		Certainty	Importance
№ of Studies	Study Design	Risk of Bias	Inconsistency	Indirectness	Imprecision	Other Considerations	SMART-CPT	CPT	Relative (95% CI)	Absolute (95% CI)		
1	randomised trials	serious [a]	not serious	Not serious	very serious [b]	none	51	49	-	MD **0.21 higher** (0.33 lower to 0.75 higher)	⨁◯◯◯ VERY LOW	

CI: Confidence interval; **MD:** Mean difference

Explanations

a. detection, performance and reporting bias

b. sample size<300

Supplementary Table S6. Prolonged exposure therapy compared to paroxetine for PTSD.

Certainty Assessment							№ of Patients		Effect		Certainty	Importance
№ of Studies	Study Design	Risk of Bias	Inconsistency	Indirectness	Imprecision	Other Considerations	PE	PH	Relative (95% CI)	Absolute (95% CI)		
**PTSD Symptoms Remission (PDS)**												
1	randomised trials	serious [a]	not serious	not serious	very serious [b]	none	72/110 (65.5%)	13/30 (43.3%)	**RR 1.51** (0.98 to 2.32)	**221 more per 1.000** (from 9 fewer to 572 more)	⨁◯◯◯ VERY LOW	

CI: Confidence interval; **RR:** Risk ratio

Explanations

a. performance and detection bias, incomplete outcome data

b. sample size < 300 patients

Supplementary Table S7. Cognitive therapy compared to repeated assessments for PTSD.

Certainty Assessment							№ of Patients		Effect		Certainty	Importance
№ of Studies	Study Design	Risk of Bias	Inconsistency	Indirectness	Imprecision	Other Considerations	CS	RA	Relative (95% CI)	Absolute (95% CI)		
**Symptoms Severity Changes (PDS Frequency)**												
1	randomised trials	serious [a]	not serious	not serious	very serious [a]	none	28	26	-	MD **14.3 lower** (20.05 lower to 8.55 lower)	⨁◯◯◯ VERY LOW	
**Symptoms Severity Changes (PDS Distress)**												
1	randomised trials	serious [a]	not serious	not serious	very serious [a]	none	28	26	-	MD **12.5 lower** (18.55 lower to 6.45 lower)	⨁◯◯◯ VERY LOW	
**Symptoms Severity Changes (CAPS-SX Frequency)**												
1	randomised trials	serious [a]	not serious	not serious	very serious [a]	none	28	26	-	MD **14.4 lower** (20.66 lower to 8.14 lower)	⨁◯◯◯ VERY LOW	
**Symptoms Severity Changes (CAPS-SX Intensity)**												
1	randomised trials	serious [a]	not serious	not serious	very serious [a]	none	28	26	-	MD **12.2 lower** (17.95 lower to 6.45 lower)	⨁◯◯◯ VERY LOW	
**Disability (Sheehan Disability Scale)**												
1	randomised trials	serious [a]	not serious	not serious	very serious [a]	none	28	26	-	MD **1.9 lower** (3.17 lower to 0.63 lower)	⨁◯◯◯ VERY LOW	
**Disability (CAPS Disability)**												
1	randomised trials	serious [a]	not serious	not serious	very serious [a]	none	28	26	-	MD **0.7 lower** (1.1 lower to 0.3 lower)	⨁◯◯◯ VERY LOW	

CI: Confidence interval; **MD:** Mean difference

Explanations

a. Performance bias and detection bias
b. sample size <300 patients
